# Combination of scoring schemes for protein docking

**DOI:** 10.1186/1471-2105-8-279

**Published:** 2007-08-01

**Authors:** Philipp Heuser, Dietmar Schomburg

**Affiliations:** 1 Cologne University Bioinformatics Center (CUBIC), University of Cologne, Zuelpicher Str. 47, 50674 Koeln, Germany

## Abstract

**Background:**

Docking algorithms are developed to predict in which orientation two proteins are likely to bind under natural conditions. The currently used methods usually consist of a sampling step followed by a scoring step. We developed a weighted geometric correlation based on optimised atom specific weighting factors and combined them with our previously published amino acid specific scoring and with a comprehensive SVM-based scoring function.

**Results:**

The scoring with the atom specific weighting factors yields better results than the amino acid specific scoring. In combination with SVM-based scoring functions the percentage of complexes for which a near native structure can be predicted within the top 100 ranks increased from 14% with the geometric scoring to 54% with the combination of all scoring functions. Especially for the enzyme-inhibitor complexes the results of the ranking are excellent. For half of these complexes a near-native structure can be predicted within the first 10 proposed structures and for more than 86% of all enzyme-inhibitor complexes within the first 50 predicted structures.

**Conclusion:**

We were able to develop a combination of different scoring schemes which considers a series of previously described and some new scoring criteria yielding a remarkable improvement of prediction quality.

## Background

Protein-protein interactions and complex formation play a central role in a broad range of biological processes, including hormone-receptor binding, protease inhibition, antibody-antigen interaction and signal transduction [1]. As structural genomics projects proceed, we are confronted with an increasing number of proteins with a characterised 3D structure but without a known function. To identify how two proteins are interacting will be particularly important for elucidating functions and designing inhibitors [2]. Although predicting around 50 percent false positive interactions [3], high throughput interaction discovery methods, such as the yeast two hybrid system, suggest thousands of protein-protein interactions and therefore also imply that a large fraction of all proteins interact with other proteins [4].

Since many biological interactions occur in transient complexes whose structures often cannot be determined experimentally, it is important to develop computational docking methods which can predict the structure of complexes with a proper accuracy [5].

Docking algorithms are developed to predict in which orientation two proteins are likely to bind under natural conditions. They can be split in a sampling step followed by a scoring step. A collection of putative structural complexes is generated by scanning the full conformational space in the first step, taking only geometric complementarity in consideration. Afterwards the putative complexes are ranked according to scoring functions based on chemical and additional aspects of geometrical complementarity.

For the ranking and scoring of potential complex structures we previously published a method based on optimised amio acid specific weighting factors [6]. With the optimised weighting factors a weighted geometric correlation is calculated using a grid representation for the proteins. More than 90% of all near-native structures for the enzyme-inhibitor complexes are found within the top 10% of the ranked output after rescoring with the optimised grid values. For all three complex-classes (antibody-antigen, enzyme-inhibitor and 'others') the number of near-native complex structures (RMSiCα < 5 Å) within the top 100 ranks increased by a factor of 3–5.

The optimised parameters comply partially with known properties of amino acids in protein complex interfaces. Amino acids for which the optimisation produced very low weighting factors are likely to cause clashes in unbound docking especially for the near-native structures. They would be misleading for the docking of unbound proteins. The lowest values (~0) were assigned to the flexible polar amino acids such as ARG, ASP, GLN, GLU or LYS, which also have a very low interface propensity [7]. For the the aromatic residues high values were obtained which comply with the ability of their ring-systems to form π-stacks and with their high propensity [7] to be in interface regions, together with the rigidity of the aromatic ring system.

Neuvirth et al. [8] reported different interface propensities for different atom-types, and there are specific atoms which play a crucial role in interactions, e.g. by their ability to participate in H-bonds. To take these phenomena into account, we also optimised atom specific weighting factors for the scoring of complex structures and evaluated their ability to identifiy near-native structures. Furthermore the combination of the amino acid specfic scoring with the atom specific scoring is evaluated.

There are other scoring functions available, which consider atoms for the scoring of predicted protein-protein complex structures, especially such that are based on knowledge-based atom-atom potentials (e.g. [9-11]). There are two main differences between this approach and atom-atom contact potentials. The parameters optimised here are derived from complex structures docked with unbound proteins whilst most atom-atom potentials were derived from native structures. Deriving the parameters from unbound structures enables us to consider atom-specific clash probabilities. This became already obvious from the optimised amino acid specific parameters where the flexibility of the amino acids influenced the weighting factors [6]. The other difference is that there is only one weighting factor for each atom type, being independent from the atom type it is in contact with. The advantage here is that wrong conformations of side chains as they appear often in rigid body unbound docking do not necessary result in loosing the contribution of these atoms towards the score.

As already described for the optimisation of the amino acid specific factors, we optimised the atom specific weighting factors for antibody-antigen, enzyme-inhibitor and 'other' complexes following the classification of the docking-benchmark2.0 [12]. Since the optimisation of a factor for each atom-type being present in proteins would have exceeded our computational resources we used the well established atom classification system by Melo et al. [13] consisting of 40 distinct atom types. The optimisation was accomplished using the nonlinear minimisation method (nlm) from the R-package for statistical computing [14].

In parallel to the development of the optimised weighting factors a very successfull comprehensive SVM-based scoring function was developed in our group and is described elsewhere (Martin O. and Schomburg D.; Efficient Comprehensive Scoring of Docked Protein Complexes using Probabilistic Support Vector Machines; submitted 2007) [15]. For this scoring function a support vector machine was trained to combine several scoring functions which were described to be able to identify near-native complexes (e.g. specialised energy functions, evolutionary relationship, class specific residue interface propensities, gap volume, buried surface area, empiric pair potentials on residue and atom level as well as measures for the tightness of fit). The application of the SVM-based scoring function leads to a remarkable improvement of the prediction quality as shown in table 2.

**Table 2:** Combination of scoring schemes

	**unopt**	**amino acid × atom specific**	**SVM**	**AA × ATM & SVM**	**unopt**	**amino acid × atom specific**	**SVM**	**AA × ATM & SVM**	**unopt**	**amino acid × atom specific**	**SVM**	**AA × ATM & SVM**
**Validation (Literature-data, 12°)**												

	**Enzyme-Inhibitor (21)**				**Antibody-Antigen (4)**				**Others (4)**			
			
**No on 1**	1	4	1	**5**	0	0	**1**	0	**1**	0	**1**	0
**No = 10**	2	7	7	**13**	0	0	**1**	0	**1**	0	**1**	0
**No = 50**	6	12	**16**	15	1	0	**2**	**2**	**1**	0	**1**	0
**No = 100**	8	13	**18**	17	1	0	**2**	**2**	**1**	0	**1**	0
**Average**	706	606	58	**55**	9887	442	299	**115**	3493	4188	**1978**	2701

**Validation (Benchmark 2.0, 12°)**												

	**Enzyme-Inhibitor (22)**				**Antibody-Antigen (19)**				**Others (29)**			
			
**No on 1**	0	2	**4**	3	0	1	0	**1**	0	0	0	**1**
**No = 10**	0	5	**11**	**11**	1	2	3	**4**	1	4	0	**6**
**No = 50**	1	12	18	**19**	1	3	7	**10**	2	**7**	5	**7**
**No = 100**	7	15	18	**19**	1	7	9	**12**	3	9	7	**12**
**Average**	897	416	113	**104**	5393	1245	350	**302**	4846	1219	1117	**712**

Number of complexes for which a near-native structure can be predicted on the first and within the top10, top50 and top100 ranks. Furthermore the average rank of the first near-native structure is given. These quality measurements are shown for an non-optimised ranking, based on the geometric correlation (unopt), a ranking based on the combination of amino-acid specific and atom specific weighting factors (amino acid × atom specific), a ranking based on SVM scoring (SVM), and for the combination of amino acid, atom specific and SVM-based ranking AA × ATM & SVM. The combination of all three scoring schemes for enzyme-inhibitor complexes and for antibody-antigen complexes is done by ranking all structures by the SVM score and than ranking all structures with the same score again by the weighted geometric correlation. For the 'other' complexes the combination is done by multiplying all scores.

However, there is no factor included in this scoring function which directly describes the geometric fit of the two binding proteins. Thus we also show the results of the combination of the SVM-based scoring function with the scoring based on the weighted geometric correlation.

## Results

### Atom specific weighting factors

**Figure 1:**
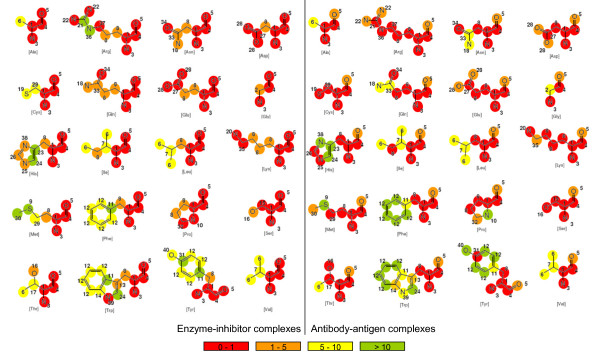
**Optimised atom specific weighting factors**. Colour coded atom specific weighted factors mapped on the 2D structures of the amino acids for enzyme-inhibitor and antibody-antigen complexes. The corresponding figure for 'other' complexes can be found within the supplementary material (additional file 2: supp_atm_factors_other.pdf). The numbers next to the atoms indicate the atom class.

The weighting factor for each atom class and the estimated value for all cells of the interior of the receptor obtained by the optimisation are shown for the three different complex classes in the supplementary material (additional file 1: supp_table_atm_factors.pdf). For visualisation purposes the weighting factors were divided in 4 classes (very low: 0–1, low: 1–5, high: 5–10 and very high: >10) and mapped in different colours on the corresponding 2-D amino acid structures (figure 1 for enzyme-inhibitor and antibody-antigen complexes and a figure for the 'other' complexes as additional file 2: supp_atm_factors_oth.pdf).

The atoms of the backbone were assigned very low (~0) values for all three complex classes except for the oxygens in antibody-antigen complexes (1.28). Atoms that are part of aromatic ring-systems were allocated high to very high values in all three classes.

For most of the other atom types the obtained parameters differ between the three complex classes. For antibody-antigen and for the 'other' complexes especially atoms that can be part of a hydrogen bond got higher values, while atoms that mainly contribute to the shape of the interface, like the methylene groups of the longer side-chains got low and very low values.

Especially for those atoms which are only present in the short side chains of ILE, LEU and VAL the optimisation yielded higher values for enzyme-inhibitor complexes than for the antibody-antigen complexes.

The values optimised for the interior of the receptor (I1) slightly differ for the three classes. For enzyme-inhibitor complexes the value is 0.67, for antibody-antigen complexes -0.87 and for the 'other' complexes 0.70.

### Improvement of prediction quality

**Figure 2:**
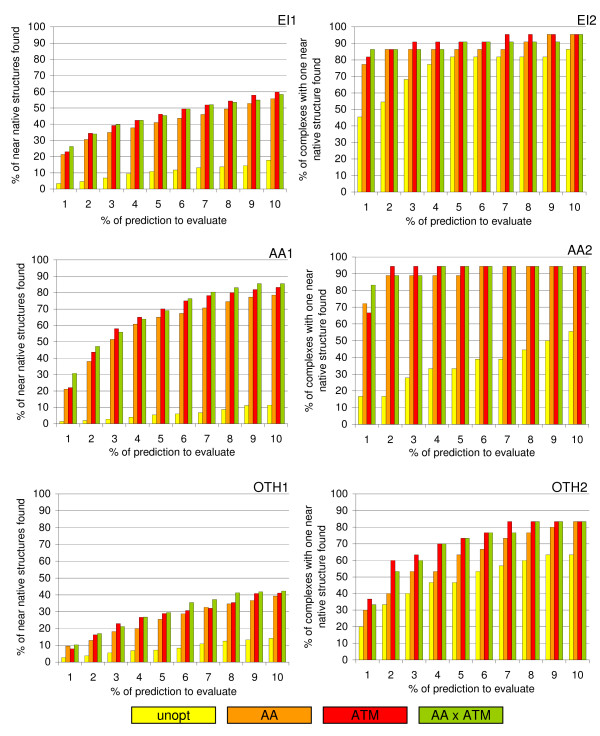
**Enrichment of near-native structures within the top 10% of the prediction **for the amino acid specific ranking (AA), the atom specific ranking (ATM) and for the combination of both (AA × ATM) compared to the non-optimised values (unopt). The enrichment is shown with the respect to the percentage of near-native structures (EI1, AA1, OTH1) and with respect to the percentage of complexes which show at least one near native structure within the top 10% (EI2, AA2, OTH2). EI1/2 for enzyme-inhibitor complexes, AA1/2 for antibody-antigen complexes and OTH1/2 for the 'other' complexes.

The results of the application of the atom specific weighting factors is shown in table 1 and in figure 2. Figure 2 illustrates the strong enrichment of near native structures within the top 10% of the sorted prediction and in table 1 the number of complexes is shown for which a near native structure is found on the first rank and within the top10, top50, and top100 ranks. Furthermore the average rank for the first near-native structure (RMSD of the interface Cα atoms below 5 Å) for each complex is given. Table 1 and figure 2 both show the improvement of prediction quality, compared to the results obtained from a purely geometric ranking, for the reranking based on the previously published amino acid specific weighting factors, for the atom specific weighting factors, and for a ranking based on the arithmetic mean of amino acid and atom specific scores.

**Table 1:** Improvement of prediction quality due to atom specific weighting

	**unopt**	**amino acid specific**	**atom specific**	**amino acid × atom specific**	**unopt**	**amino acid specific**	**atom specific**	**amino acid × atom specific**	**unopt**	**amino acid specific**	**atom specific**	**amino acid × atom specific**
**Optimisation (Benchmark 2.0, 15°)**												

	**Enzyme-Inhibitor (22)**				**Antibody-Antigen (18)**				**Others (30)**			
			
**No on 1**	**0**	**1**	3	3	**0**	1	1	1	**0**	**0**	**0**	**0**
**No = 10**	2	5	6	**9**	1	1	**3**	**3**	0	**2**	1	**1**
**No = 50**	4	12	13	**14**	1	3	**6**	5	1	4	**5**	**5**
**No = 100**	6	15	**16**	**16**	2	**9**	8	8	2	6	**8**	7
**Average**	1222	370	**242**	283	3119	462	**267**	295	3088	1560	**1303**	1323

**Validation (Literature-data, 12°)**												

	**Enzyme-Inhibitor (21)**				**Antibody-Antigen (4)**				**Others (4)**			
			
**No on 1**	1	1	1	**4**	0	0	0	0	**1**	0	0	0
**No = 10**	2	5	**7**	**7**	0	0	0	0	**1**	0	0	0
**No = 50**	6	10	**14**	12	**1**	0	0	0	**1**	0	0	0
**No = 100**	8	12	**14**	13	**1**	0	0	0	**1**	0	0	0
**Average**	918	720	669	**606**	9887	568	499	**442**	**3493**	5761	3513	4188

Number of complexes for which a near-native structure can be predicted on the first and within the top10, top50 and top100 ranks. Furthermore the average rank of the first near-native structure is given. These quality measurements are shown for a non-optimised ranking, based on the geometric correlation (unopt), a ranking based on the amino-acid specific weighting factors (amino acid specific), a ranking based on the atom specific weighting factors (atom specific), and for the combination of amino acid and atom specific ranking.

The results achieved by the application of the atom specific weighting factors are comparable to those results which can be achieved with the amino acid specific scoring. Depending on the way of quality measurement the atom specific reranking is slightly better than the amino acid specific weighting. Especially the number of complexes for which a near native structure can be found on the top ranks (table 1) is higher with the atom specific factors and the average rank of the first near native structure is considerably lower.

For the four antibody-antigen and for the four 'other' complexes in the validation set the performance of the weighted scoring is worse than the purely geometric scoring with respect to the number of complexes with a near native prediction on the top ranks. However for both of them the average rank of the first near native structure is also considerably lower with the atom specific scoring than with the amino acid specific scoring.

The results obtained by a scoring with the mean of amino acid and atom specific scores are nearly identical to those which can be obtained by the atom specific scoring. Since the enrichment of near native structures on the lower ranks (figure 1) is slightly higher with the combination of both scoring schemes this combination is used for further scorings.

**Figure 3:**
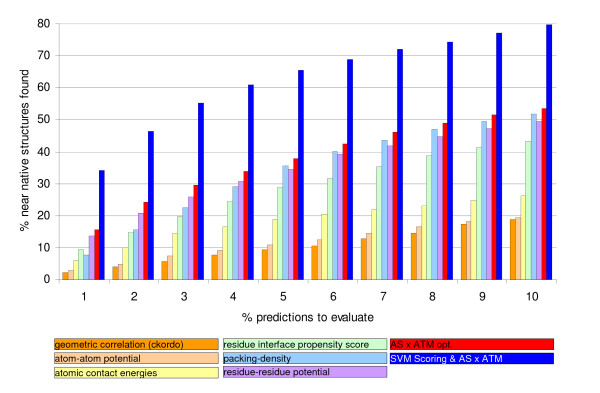
**Comparison to other scoring methods**. Enrichment of near native structures due to a ranking with the weighted geometric scoring and the combination with the SVM based scoring compared to the enrichment obtained by ranking with atomic contact energies (ACE) [25], a residue-residue potential [11], an atom-atom potential [9], the scoring function based on complex-class specific residue interface-propensity by Huang et al. [26] and the calculation of packing density [26, 27].

Figure 3 shows the enrichment of near-native structures due to a ranking with the combined atom and amino acid specific weighting factors in comparison to 5 other scoring functions used to rank potential protein complex structures.

Table 2 shows the dramatic improvement of prediction quality due to the combination of the scoring based on the weighted geometric score with the SVM based scoring by Martin [15]. The combination of both scorings further improves the results for all three complex classes. After the application of all scoring functions for 19 of the 22 enzyme-inhibitor complexes, for 12 of the 19 antibody-antigen complexes and for 12 of the 29 'other' complexes a near native structure can be found within the top 100 ranks. Altogether the percentage of complexes for which a near native structure can be predicted within the top 100 ranks increased from 14% with the geometric scoring to 54% with the combination of all scoring functions.!

## Discussion

The combination of both scoring schemes – the weighted geometric correlation and the comprehensive scoring function from Martin [15] – yields a considerable improvement of prediction quality. Especially for the enzyme-inhibitor complexes the results of the ranking are excellent. For half of these complexes a near-native structure can be predicted within the first 10 proposed structures and for more than 86% of all enzyme-inhibitior complexes within the first 50 predicted structures. On average for more than 50% of all complexes a near-native structure is predicted within the top100 ranks.

However, even though most previously described scoring schemes influence the final combined ranking it is not possible to predict all complex structures reliably. Especially for the 'other' complexes the results are not satisfying. For less than 50% of the 'other' complexes a near native structure can be found within the top 100 solutions after the reranking. The reason lies most likely within the inherent heterogeneity of the 'other' complexes (different function, evolutionary restraints, chemical environment, etc.). Furthermore there is no explicit treatment of flexibility included in the docking procedure yet. Even though the optimised weighting factors improve the results when the side chain flexibility is not taken into account, they are not capable to deal with any major movements of the backbone.

The necessary different ways of combining the weighted geometric fit with the SVM scoring is based on the differing success of the SVM-based scoring for the three complex classes. While the SVM-based scoring is highly specific and sensitive for enzyme-inhibitior complexes and antibody-antigen complexes, the sensitivity and performance for the 'other' complexes is worse. Therefore the best combination of weighted geometric score and SVM-based score for enzyme-inhibitor and antibody-antigen complexes is to sort all structures with the same SVM-score again by the weighted geometrical score, while the best results for the 'other' complexes were obtained by multiplying both scores.

The results obtained by the atom specific weighting factors are slightly better than the results obtained by the amino acid specific scoring, which is probably due to the more detailed resolution of an atom specific scoring.

The obtained optimised weighting factors partially mirror the known role of different atom types in protein complex interfaces. Most remarkably are the very low values which were obtained for the backbone atoms. On the one hand these values comply with the low interface propensity for backbone atoms described by Neuvirth et al. [8] and on the other hand it is quite likely that true and false interface regions do not differ significantly with respect to the number of backbone contacts.

For all three complex classes the values assigned to the atoms of the aromatic ring systems are rather high. As already discussed for the amino acid specific weighting factors [6], this can be explained with the high interface propensity of the aromatic residues [7,16], with their ability to form intermolecular π-stacks and with their rigidity.

The other values differ considerably for the different complex classes. Whilst for the enzyme-inhibitor complexes especially such atoms got higher values which are responsible for the overall shape of the interface and which are quite rigid, for the antibody-antigen and to a certain extend for the 'other' complexes those atoms which show some functionality, e.g. participating in H-bonding, were assigned higher values reflecting the higher importance of hydrogen bonds in the binding region of the antibody.

The higher values for the shape-giving atoms, like the carbon atoms of the shorter side chains of ILE, LEU or VAL and the methylene groups of the longer side-chains, comply with the higher packing density of the enzyme-inhibitor complexes [17]. Whilst the higher values for the nitrogen and oxygen atoms of antibody-antigen complexes (e.g. in ASN, GLN, ASP, GLU, ARG, PRO and O within the backbone) can be explained by the importance of H-bonds and electrostatics for the binding of antigens and antibodies [18].

For some of the atoms, especially those that appear in multiple residues (e.g. backbone atoms or side chain carbon atoms) a more detailed atom classification might yield better results. A finer classification might consider the amino acids or the distance to the backbone. Unfortunately our computational resources would not have been sufficient for the optimisation of a higher number of parameters.

The values optimised for the interior cells of the larger protein are comparable to those optimised for the amino acid specific weighting factors [6]. The values for the enzyme-inhibitor and for the 'other' complexes are positive, while the value for the antibody antigen complexes is negative. The positive value for the enzyme-inhibitor complexes is explainable by the tight fit of the enzymes and their inhibitors. Any clashes which are present in the near-native structures are most likely caused by the flexibility of the proteins. All structures, which are part of the proposed structures for the reranking even though they have some clashes, must have an excellent overall geometrical correlation, which in turn is a property of the near-native structures.

The validation of the obtained weighting factors shows that the optimised enzyme-inhibitor factors also work as expected for complexes which were not present during the optimisation process. Even though the number of antibody-antigen complexes having a near-native structure among the top 100 is lower after the reranking the weighting factors still work for these complexes, which becomes obvious from the considerably lower average rank of the first near-native structure.

The results produced for the validation of the parameter for the 'other' complexes are not convincing, but the number of these complexes was too low for a reliable validation with respect to the heterogeneity of the 'other' complexes. Since the optimised parameters work reliably for the benchmark2.0 set docked with a 12° increment, where especially the false structures are different from the data used for the optimisation, we are still optimistic that these parameters will work for new 'other' complexes.

In comparison with some of the other known scoring functions for protein complexes the weighted geometric correlation is able to enrich more near-native structures within the top 10% of the ranked prediction as shown in figure 3. The combination of SVM-based scoring and weighted geometric correlation more than doubles the percentage of near native structures which can be found in the 1^st ^% of the ranking as compared to the other scoring functions.

## Conclusion

We were able to develop a combination of different scoring schemes yielding a remarkable improvement of prediction quality. In the SVMs most previously described scoring criteria are included. The unsatisfactory results for some complexes, even though most ranking criteria are used, demonstrate again the importance of explicit treatment of flexibility for docking. However, the ranking schemes here should work with any grid based docking scheme.

The docking procedure described in this and our previous papers will be available on the internet very soon, once we are finished with the optimisation of computer time.

## Methods

### The docking tool

In a first step we used our docking tool *ckordo*[19,20] to generate potential complex structures for 83 unbound protein-protein complexes from the Benchmark 2.0 set [12]. *Ckordo *is a FFT based docking program including further docking arguments such as hydrophobicity and electrostatics. For this work we used only the geometric correlation calculated in Fourier space. For the calculation of the geometric correlation the unbound protein structures are mapped on a 3D grid. Numerical values are assigend to the grid cells depending on their location. Cells representing the surface of the receptor and all cells of the ligand get a value of one. The interior cells of the receptor get -6 (for a more detailed description see [6]). The geometric correlation was calculated with a rotation increment of 12° and a maximum cell size of 1.5 Å. For each rotation the five structures with the highest geometrical correlation were considered. This lead to 43,080 potential structures for each complex showing a reasonable geometric fit.

Since the optimisation procedure (see below) is rather expensive with respect to memory and cpu-power, it was impossible to use 43,080 structures for each complex for the optimisation. An additional *ckordo *run was done using only 15° rotational increment leading to 22,000 structures per complex, which were used for the optimisation.

Furthermore for each proposed structure the root mean square deviation of the Cα atoms in the interface (RMSiCα) was calculated, in comparison to the unbound proteins fitted on the complex. For the RMSD calculation the Cα-atoms were defined to be part of the interface if at least one atom of the other protein was within a distance of 10 Å. The fitting of the unbound proteins on the complex was done with CE [21].

### Optimisation

In an extension of the described FFT-based docking procedure we replaced the standard numverical values by optimised atom-specific parameters.

Since the total number of near-native structures produced by a *ckordo *run is very low compared to the number of incorrect ones, additional solutions with low RMS-values were produced by running *ckordo *for 1000 randomly chosen angles in the range from -10° to +10° around the correct rotation. From this run all proposed complex structures with RMSiCα < 5 Å were selected. This resulted in up to 4,700 additional near-native solutions for each complex, which were added to the 22,000 structures calculated with rotational steps of 15°. If the ratio between the number of near-native solutions towards wrong ones is too low, the optimisation procedure would not be able to find the optimal parameters.

For the optimisation process *ckordo *was modified so that for each proposed structure the number of contacts of each atom type with respect to being surface or interior was calculated. This results in one 40 × 40 matrix for each structure for each possible contact type (surface_protein1 × protein2, interior_protein1 × protein2).

For the optimisation we used the same procedure as for optimising the amino acid specific weighting factors [6]. The contact-matrices and the RMS-values for the complexes from the Benchmark 2.0 [12] were used for the optimisation procedure. For each complex class the optimisation was performed independently. The optimisation was done using the nonlinear minimisation method (nlm) from the R-package for statistical computing [14].

The optimisation itself is a minimisation of the quadratic error between an objective function and the scores obtained. For the objective function all near-native structures (RMSiCα: 0–5 Å) were assigned a 100 times higher numerical value (10,000) as those showing a RMS value higher than 10 Å. For those structures between near-native and 'wrong' structures (RMSiCα: 5–10 Å) the target values were calculated by a linear function.

The task for the optimisation was to find suitable weighting factors for each atom type, such that the calculated score for each potential complex structure gets as close as possible to the desired values (i.e. high for near-native structures and low for false structures).

Since the inclusion of the value for the interior of the larger protein in the optimisation yielded better results for the amino acid specific weighting factors we also included this value here.

The nonlinear minimisation function of the R-package[14] uses a Newton-type algorithm [22,23]. This method allows finding a minimum of a function by numerical computation of the derivatives. As a convergence criterion for the optimisation the default parameters were used.

### Validation

Due to hardware limits it was impossible to use all available structures for the optimisation, so that subsets had to be chosen. To prove that several different subsets lead to similar results a 5-fold cross validation procedure was performed. Therefore the different complexes from each class were grouped randomly in 5 groups. The optimisation was run 5 times each time leaving out one of the groups and optimising with the remaining four. The final results were calculated using the average value of the five optimisations.

Furthermore the effect of the obtained parameters was evaluated on 21 enzyme-inhibitor, 4 antibody-antigen and 4 'other' complexes from literature [24], which were not part of the training. These test-cases are identical to those used for validation in Heuser & Schomburg 2006[6]. The docking procedure for these test cases was run with a rotation increment of 12° leading to 43080 potential structures for each complex. The evaluation was done with respect to the number of complexes which do have a near-native solution within the top ranks and to the number of near-native structures on the first ranks.

In addition the ranking schemes were tested on the same complexes which were used for the optimisation, but docked with 12° increment. This leads to a different set of proposed structures. Especially the false structures are different to the false structures used for the training.

The ability of the combined scoring of atom and amino acid specific weighting factors to enrich near native structures within the top 10% of the ranking is compared to 5 other scoring methods. The other scoring methods used are atomic contact energies (ACE) [25], a residue-residue potential [11], an atom-atom potential[9], the scoring function based on complex-class specific residue interface-propensity by Huang et al. [26] and the calculation of packing density. [26,27]

### Reranking

For the reranking the common grid representation as used in ckordo is extended by the weighting factor. Each cell representing the protein gets a value assigned, which is the product of the value for being surface or interior and the optimised weighting factor. To obtain the weighted geometric correlation the values of the overlapping cells are multiplied in the same way as it is done for the calculation of the geometric correlation.

For the combination of atom and amino acid specific scores a ranking based on the geometric and arithmetic mean of both scores was evaluated where the latter yielded better results.

The combination of SVM-based scoring[15] and weighted geometric correlation was done in three different ways. On the one hand the ranking ability of the geometric and arithmetic mean of both scores was evaluated. On the other hand, since the SVM-based scoring classifies many of the potential structures for enzyme-inhibitor and antibody-antigen complexes as being near-native, it was tried to use the SVM-based ranking and rerank all those structures which obtained the same score from the SVM ranking once again with the weighted geometric correlation. The latter turned out to yield best results for enzyme-inhibitor and antibody-antigen complexes, while the arithmetic mean of atom specific, amino acid specific, and SVM based scores produced the best results for the 'other' complexes.

## Authors' contributions

The described results are obtained by a combined effort of the two authors where PH did the actual computer work including programming and integration of the method whereas the method was developed and results were discussed in frequent discussions between the two authors. The original idea was from DS.

## Supplementary Material

Additional file 1 Table with the optimised atom specific weighting factors for all 40 atom types for enzyme-inhiobitor, antibody-antigen and 'other' complexes and their standard deviation as derived from the 5-fold crossvalidation. Click here for file

Additional file 2 Colour coded atom specific weighted factors mapped on the 2D structures of the amino acids for 'other' complexes. Click here for file

## References

[B1] Valdar WS, Thornton JM (2001). Protein-protein interfaces: analysis of amino acid conservation in homodimers. Proteins.

[B2] Caffrey P (2003). Conserved amino acid residues correlating with ketoreductase stereospecificity in modular polyketide synthases. Chembiochem.

[B3] Deane CM, Salwinski L, Xenarios I, Eisenberg D (2002). Protein interactions: two methods for assessment of the reliability of high throughput observations. Mol Cell Proteomics.

[B4] Aloy P, Russell RB (2002). The third dimension for protein interactions and complexes. Trends Biochem Sci.

[B5] Halperin I, Ma B, Wolfson H, Nussinov R (2002). Principles of docking: An overview of search algorithms and a guide to scoring functions. Proteins.

[B6] Heuser P, Schomburg D (2006). Optimised amino acid specific weighting factors for unbound protein docking. BMC Bioinformatics.

[B7] Chakrabarti P, Janin J (2002). Dissecting protein-protein recognition sites. Proteins.

[B8] Neuvirth H, Raz R, Schreiber G (2004). ProMate: a structure based prediction program to identify the location of protein-protein binding sites. J Mol Biol.

[B9] Grimm V (2003). Untersuchung eines wissensbasierten Potentials zur Bewertung von Protein-Protein-Docking-Studien.. Institut für Biochemie.

[B10] Kozakov D, Brenke R, Comeau SR, Vajda S (2006). PIPER: an FFT-based protein docking program with pairwise potentials. Proteins.

[B11] Moont G, Gabb HA, Sternberg MJ (1999). Use of pair potentials across protein interfaces in screening predicted docked complexes. Proteins.

[B12] Mintseris J, Wiehe K, Pierce B, Anderson R, Chen R, Janin J, Weng Z (2005). Protein-Protein Docking Benchmark 2.0: an update. Proteins.

[B13] Melo F, Feytmans E (1997). Novel knowledge-based mean force potential at atomic level. J Mol Biol.

[B14] R Development Core Team (2005). R: A language and environment for statistical computing.

[B15] Martin O (2006). Effcient comprehensive scoring of docked protein complexes - a machine learning approach. Instutute for Biochemistry/Cologne University Bioinformatics Center (CUBIC).

[B16] Lo Conte L, Chothia C, Janin J (1999). The atomic structure of protein-protein recognition sites. J Mol Biol.

[B17] Jones S, Thornton JM, Kleanthous C (2000). Analysis and classification of protein-protein interactions from a structural perspective. Protein-Protein Recognition.

[B18] Jackson RM (1999). Comparison of protein-protein interactions in serine protease-inhibitor and antibody-antigen complexes: implications for the protein docking problem. Protein Sci.

[B19] Meyer M, Wilson P, Schomburg D (1996). Hydrogen bonding and molecular surface shape complementarity as a basis for protein docking. J Mol Biol.

[B20] Zimmermann O (2002). Untersuchungen zur Vorhersage der nativen Orientierung von Protein-Komplexen mit Fourier-Korrelationsmethoden. Institute for Biochemistry.

[B21] Shindyalov IN, Bourne PE (1998). Protein structure alignment by incremental combinatorial extension (CE) of the optimal path. Protein Eng.

[B22] Dennis JE, Schnabel RB (1983). Numerical Methods for Unconstrained Optimization and Nonlinear Equations. Prentice-Hall, Englewood Cliffs, NJ.

[B23] Schnabel RB (1985). A modular system of algorithms for unconstrained minimization.. ACM Trans Math Software.

[B24] Heuser P, Martin O UUPPDD - Unbound Unbound Protein Protein Docking Dataset. http://biotool.uni-koeln.de/uuppdd/.

[B25] Zhang C, Vasmatzis G, Cornette JL, DeLisi C (1997). Determination of atomic desolvation energies from the structures of crystallized proteins. J Mol Biol.

[B26] Huang B, Schroeder M, Matthias R, Andrew T, Kurtz S, Willhoeft U (2005). Using residue propensities and tightness of fit to improve rigid-body protein-protein docking.. Proceedings of the German Conference on Bioinformatics (GCB2005), Hamburg, Germany, October 5-7, 2005.

[B27] Gottschalk KE, Neuvirth H, Schreiber G (2004). A novel method for scoring of docked protein complexes using predicted protein-protein binding sites. Protein Eng Des Sel.

