# Supplementary material for: Combination of scoring schemes for protein docking
Source: BMC Bioinformatics. 2007 Aug 1;8:279. doi: 10.1186/1471-2105-8-279 (PMC1978211; doi:10.1186/1471-2105-8-279)
Supplement: Additional file 1 — Table with the optimised atom specific weighting factors for all 40 atom types for enzyme-inhiobitor, antibody-antigen and 'other' complexes and their standard deviation as derived from the 5-fold crossvalidation. [file 1471-2105-8-279-S1.pdf]

atom-class	antibody-antigen	std.-dev.	enzyme-inhibitor	std.-dev.	others	std.-dev.
1	**0,00**	0,00	**0,01**	0,01	**0,00**	0,00
2	**8,62**	3,45	**3,30**	1,78	**14,88**	2,65
3	**0,00**	0,00	**0,04**	0,04	**0,00**	0,00
4	**0,01**	0,00	**0,01**	0,01	**0,00**	0,00
5	**1,28**	0,85	**0,07**	0,09	**0,00**	0,00
6	**7,50**	0,39	**6,18**	0,95	**3,75**	0,83
7	**6,52**	1,37	**6,20**	2,96	**9,52**	4,01
8	**0,18**	0,28	**2,61**	0,32	**0,00**	0,00
9	**10,08**	8,49	**25,77**	8,78	**5,59**	6,05
10	**4,78**	2,12	**0,07**	0,03	**11,03**	9,92
11	**9,21**	9,07	**19,61**	7,27	**17,64**	12,79
12	**10,00**	1,48	**8,81**	1,12	**8,76**	2,20
13	**3,51**	4,17	**2,08**	3,28	**0,02**	0,03
14	**1,93**	2,96	**6,81**	7,19	**11,03**	11,03
15	**0,00**	0,00	**0,00**	0,01	**3,14**	2,71
16	**0,00**	0,00	**1,83**	1,64	**6,88**	1,77
17	**0,00**	0,00	**6,29**	1,18	**2,90**	2,81
18	**5,31**	3,16	**2,28**	1,19	**0,00**	0,00
19	**0,34**	0,33	**5,28**	5,37	**5,72**	7,51
20	**0,24**	0,38	**0,67**	0,75	**0,00**	0,00
21	**0,01**	0,00	**11,61**	5,29	**0,01**	0,02
22	**1,18**	1,86	**0,01**	0,00	**6,29**	2,45
23	**11,29**	5,60	**25,18**	13,60	**1,68**	2,57
24	**19,60**	5,97	**14,26**	5,46	**0,00**	0,00
25	**0,73**	0,93	**1,56**	2,19	**2,90**	3,88
26	**0,03**	0,03	**2,79**	1,98	**0,01**	0,01
27	**0,26**	0,40	**0,01**	0,01	**0,00**	0,00
28	**3,31**	1,37	**0,02**	0,01	**0,00**	0,00
29	**0,05**	0,05	**5,18**	4,22	**0,00**	0,00
30	**1,84**	2,00	**16,88**	4,50	**11,23**	5,73
31	**0,18**	0,24	**30,84**	5,48	**8,21**	6,79
32	**4,91**	2,02	**0,05**	0,03	**0,11**	0,22
33	**5,66**	0,99	**1,32**	1,51	**6,80**	2,73
34	**0,95**	1,49	**0,01**	0,01	**9,00**	5,60
35	**1,22**	1,33	**0,01**	0,00	**9,92**	2,99
36	**0,04**	0,05	**10,91**	4,36	**1,56**	1,53
37	**0,03**	0,04	**0,30**	0,33	**0,00**	0,00
38	**20,75**	7,43	**1,61**	2,39	**3,15**	4,17
39	**8,46**	5,35	**0,03**	0,03	**0,00**	0,00
40	**23,18**	1,49	**5,46**	1,39	**18,08**	7,25
I1	**-0,87**	0,29	**0,67**	0,57	**0,70**	0,28

Optimised atom specific weighting factors

Optimised atom specific weighting factors for the 40 atom-types defined by Melo *et al.*[10] for all three complex classes and the standard deviation as derived from the 5-fold crossvalidation.
