# Supplementary material for: Combination of scoring schemes for protein docking
Source: BMC Bioinformatics. 2007 Aug 1;8:279. doi: 10.1186/1471-2105-8-279 (PMC1978211; doi:10.1186/1471-2105-8-279)
Supplement: Additional file 2 — Colour coded atom specific weighted factors mapped on the 2D structures of the amino acids for 'other' complexes. [file 1471-2105-8-279-S2.pdf]

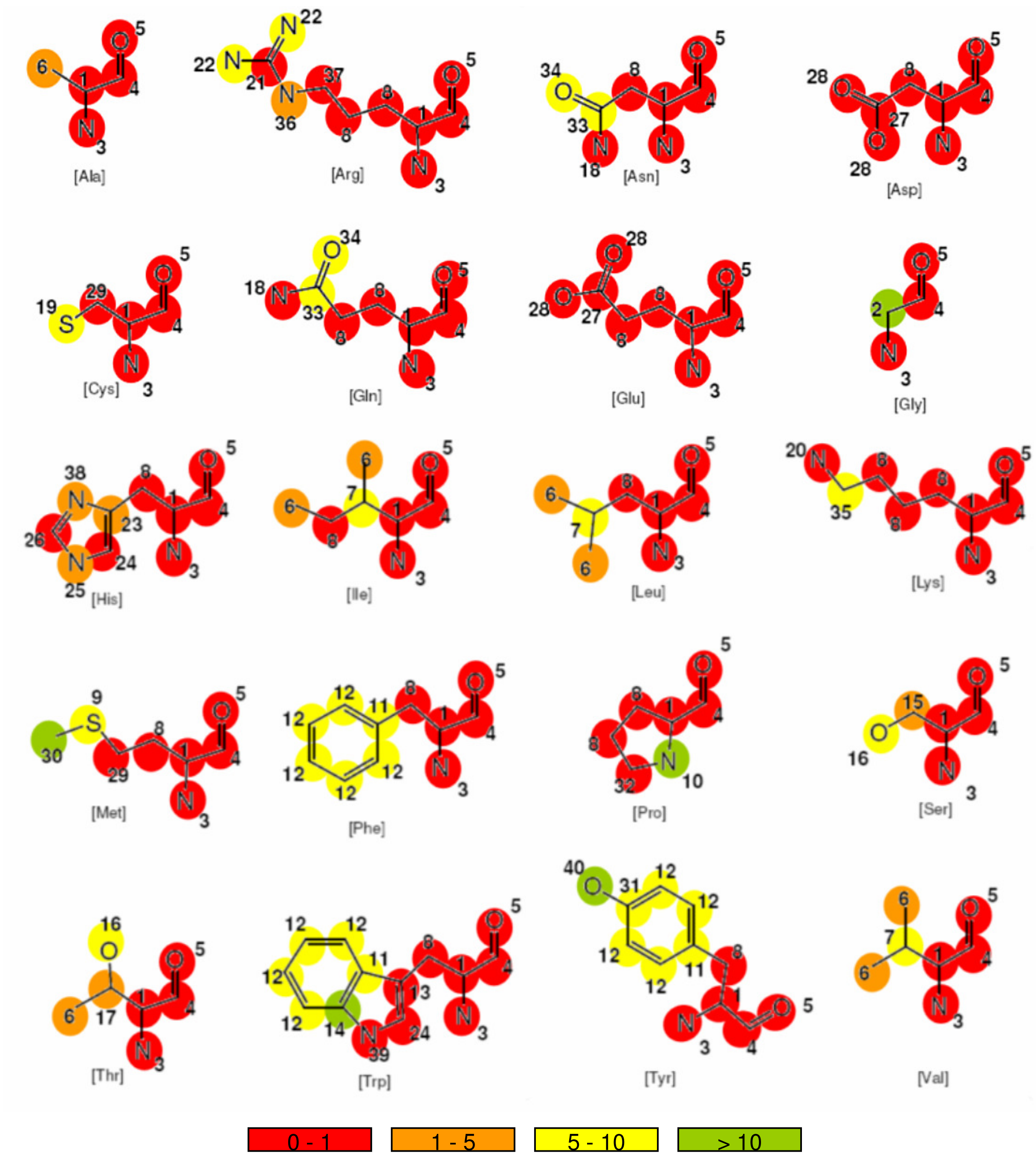

Optimised atom specific weighting factors

Colour coded atom specific weighted factors mapped on the 2D structures of the amino acids for other complexes (the numbers next to the atoms indicate the atom class)
